# Assessing patients at risk of symptomatic-but-as-yet-undiagnosed cancer in primary care using information from patient records

**DOI:** 10.1038/s41416-020-0828-4

**Published:** 2020-04-15

**Authors:** Georgios Lyratzopoulos, Gary A. Abel

**Affiliations:** 1 Epidemiology of Cancer Healthcare and Outcomes (ECHO) Group, Department of Behavioural Science and Health, Institute of Epidemiology and Health Care, University College London, 1-19 Torrington Place, London, WC1E 7HB UK; 2 University of Exeter Medical School, Smeall Building, St. Luke’s Campus, Exeter, EX1 2 LU UK

**Keywords:** Signs and symptoms

## Abstract

Evidence arising from primary care electronic health records can help to assess the risk of symptomatic-but-as-yet-undiagnosed cancer. Existing evidence and methodological innovations in this field of study hold further promise for improving the diagnostic process and achieving earlier diagnosis in cancer patients.

## Main

Most cancer patients are diagnosed after the onset of symptoms relating to their cancer.^1^ Timely assessment of patients with symptomatic-but-as-yet-undiagnosed cancer can shorten intervals to diagnosis, helping to improve clinical outcomes and patient experience.^2,3^ Sustained research efforts in understanding tumour biology have thus far not delivered the array of accurate, easily useable and cost-effective tests needed to support the diagnostic process in patients with possible cancer symptoms—most of whom will not have cancer. New tests are particularly needed to assist the diagnosis of ‘harder-to-suspect’ cancers, which most often present with symptoms of relatively low predictive value.^4^ These realisations have, increasingly since 2006,^5^ focussed research efforts on the understanding of the phenomic signature of symptomatic-but-as-yet-undiagnosed cancer using information included in ‘real-world’ electronic patient records data.

The *British* *Journal* *of Cancer* has recently published two studies of relevance to this expanding field. Nicholson et al.^6^ describe the risk of cancer diagnosis following recorded weight loss in primary care consultations; they estimate this risk to be 1.2% by the end of a 3-month follow-up period, increasing to 1.8% by 12 months. Watson et al.^7^ examined the risk of cancer following tests for inflammatory markers (such as erythrocyte sedimentation rate, C-reactive protein and plasma viscosity) in primary care; they estimate the risk to be 3.5% in patients with raised values by 12 months. Together with other studies estimating the predictive value of different symptoms and pre-diagnostic features, these estimates can support decision making at the clinical encounter and the production of clinical practice guidelines.^8^ Beyond substantially enriching current knowledge, both studies highlight broader methodological issues pertinent to the estimation of the risk of cancer diagnosis based on information included in primary care electronic patient records.

Accurate measurement of exposure is a pre-requisite for any epidemiological study, and variation between doctors in the recording of possible cancer symptoms has long been described.^9^ As clinicians may record symptom information in free-text notes differentially between cases and controls, risk estimates, particularly for ‘alarm’ symptoms with known relatively high predictive value for cancer,^10^ may be artefactually inflated.^11^ Nicholson et al.^12^ should be commended for having previously validated symptom codes associated with unintended weight loss in Clinical Practice Research Datalink data against objectively measured loss of weight in the same data source. Further, the authors of both commented studies appropriately compared the characteristics of patients with and without recorded weight or recorded inflammatory marker tests, as such analyses can help to assess the generalisability of their findings.^6,7^

Unlike information about the presence/absence of symptoms, information about the results of diagnostic tests could additionally allow for appreciation of ‘dose–response’ associations (e.g. using exact inflammatory marker,^7^ or platelet count,^13^ values), which could enable further individualising of risk estimates. Incorporating quantification of unintended weight loss in prediction models might be possible in the future, if the recording of patients’ weight as part of primary care encounters was to become uniform and systematic.^14^ Additionally, qualifying the nature (e.g. the intensity, persistence and duration) of presenting symptoms (such as weight loss) may be revealing given that they typically represent heterogeneous constructs. Nonetheless, the epidemiology of presenting symptoms in patients subsequently diagnosed with cancer remains understudied, and an area where qualitative inquiries are also needed to illuminate both the nature of symptoms and how they are appraised by patients.^15,16^

It has long been described that the risk of cancer after the first occurrence of relevant pre-diagnosed features is concentrated in the initial period post-presentation, declining rapidly thereafter.^17^ Nonetheless, in prior literature predictive value estimates are generally ‘averaged out’ over relatively lengthy periods between first exposure and cancer diagnosis, of up to 1, 2 or 3 years.^17,18^ Faced with this ‘still picture vs. video’ problem,^19^ Nicholson et al.^6^ provide time-dependent risk estimates for cancer diagnosis following consultations with unintended weight loss as a presenting symptom. They report that the hazard of cancer diagnosis was highest within the first 3 months of follow-up after this symptom was recorded, waning within 6 months or longer. The authors rightly argue that more accurately appreciation of  the evolving levels of risk after the initial recording of a given feature could be useful.

The translation of information on the time-dependent nature of risk is however not straightforward, as the evidence relates to observational data from patient care records. Therefore, the time between symptom onset and diagnosis is influenced by both the natural history of the disease and decisions about investigative management (the latter also being influenced by the presence/absence of practice recommendations included in clinical guidelines). Most cancer patients who presented with unexplained weight loss were diagnosed during the few months after this symptom was recorded.^6^ Consequently, the potential to expedite the diagnosis of these patients may be limited, although it may be possible to improve their diagnostic pathways (e.g. reducing the risk of diagnosis of cancer through an emergency presentation, a diagnostic route associated with worse prognosis^1^). There is the potential that watchful waiting, as discussed by the authors, may lead to use of fewer tests and referrals, and thus longer intervals to diagnosis and potentially worse clinical outcomes in this patient group. Symptoms associated with cancer arising long before a diagnosis is subsequently made present the best opportunity to improve the diagnostic process, but it is not obvious how these could be detected in observational studies when both natural disease progression and investigative management vary widely between patients. Further research is required to understand the degree to which the time-dependent nature of risk reflects the natural history of the disease or historical clinical practice, and how such appreciations can inform improvement efforts.

As different cancer sites have heterogeneous associations with the same pre-diagnostic feature, both reviewed studies usefully report data on the cancer site case-mix of patients with either unintended loss of weight or positive inflammatory marker testing.^6,7^ Nicholson et al.^6^ also report that, among patients with unintended weight loss, the greatest cancer site-specific risks relate to pancreatic cancer and cancer of unknown primary, while the risks of either prostate or breast cancer are lower than that in patients without unintended weight loss. Such analyses, of the ‘cancer signature’ of different pre-diagnostic features, can help to devise cost-efficient investigation strategies, individualising the cascade of specialist diagnostic tests, depending on the presenting symptoms and initial test findings.^13,20^ Echoing other recent evidence,^21^ a substantial proportion of cancer patients who presented with unintended weight loss were diagnosed in stages other than stage IV.^6^

An issue beyond the optimisation of risk prediction algorithms for symptomatic-but-as-yet-undiagnosed cancer is their appropriate and dependable incorporation into everyday clinical practice, a complex problem in need of implementation research. Artificial intelligence is considered to offer promise in improving the diagnostic process, but while it could improve risk assessment in real time, it is also associated with implementation challenges and potential safety risks.^22^ There is currently little conclusive evidence about the effectiveness of decision-support tools embedded in electronic health records, although trials are ongoing.^23,24^

A limitation of current evidence is that, with certain exceptions, studies estimate the risk of cancer without quantification of the risk of other consequential illness such as autoimmune disease or infection.^25,26^ Among patients with non-localising symptoms (such as unintended loss of weight), the yield ratio for consequential non-neoplastic illness over cancer diagnosis may be as high as 2:1.^20^ Therefore, promptly and efficiently diagnosing symptomatic-but-as-yet-undiagnosed cancer does not simply represent a problem solely for cancer research, rather one for medical research, overall. Broad coalitions of research funders, researchers, clinicians and policy-makers are needed to support research to improve the diagnosis of cancer and other significant diseases.

## Data Availability

Not applicable.
